# Removal of Fibroma during Pregnancy

**Published:** 1890-09

**Authors:** 


### REMOVAL OF FIBROMA DURING PREGNANCY.

Dr. Routier successfnlly performed this serious operation last year, pregnancy continuing undisturbed. The patient was thirty-seven years of age. The tumor appeared to have existed over six years, and there was no menorrhagia. The tumor was removed between the third and fourth months of gestation. It was a large, tuberous, kidney-shaped sub-peritoneal fibroid, over ten inches in its longest measurement. The pedicle was as thick as a man's fist. It was transfixed by two pins; the second was passed across the first, and great care was taken not to wound the uterus itself; then an elastic ligature was passed round the pedicle, and the tumor was cut away, a portion which was afterwards enucleated remaining on the cut surface of the pedicle. The pins were next removed, and the edges of the raw surface sewn together by a continuous suture of stout silk. The uterus was replaced in the abdomen, and the abdominal wound closed. The patient made a good recovery. This operation is described in the *Annales de Gynecologie et d'Obstetrique*, for March, 1890. Dr. Routier has carefully searched medical literature on the subject, and succeeded in collecting fifteen cases of removal of fibroids during pregnancy, the uterus not being amputated; of these fifteen, five died, two succumbing to hemorrhage after abortion within one week of the operation. In three out of the ten recoveries, abortion occurred; in the remaining seven pregnancy continued till term. The list includes Dr. Routier's interesting case.—*British Medical Journal.*

